# Complete genome sequence of *Vibrio fischeri* strain H905, a planktonic isolate among squid symbiotic congeners

**DOI:** 10.1128/mra.00418-24

**Published:** 2024-09-23

**Authors:** Jennifer Calawa, Randi Foxall, Sabrina Pankey, Robert Sebra, Cheryl A. Whistler

**Affiliations:** 1 Department of Molecular, Cellular, and Biomedical Science, University of New Hampshire, Durham, New Hampshire, USA; 2 Ichan School of Medicine at Mt Sinai, New York, New York, USA; Montana State University, Bozeman, Montana, USA

**Keywords:** symbiosis, *Vibrio fischeri*, bioluminescence, planktonic

## Abstract

Here we describe the genome sequence of *Vibrio* (*Aliivibrio*) *fischeri* H905, a non-symbiotic isolate from Kaneohe Bay, Hawaii. Despite its close phylogenetic relationship to squid symbiont strains, H905 is not adept at colonization. Its genome serves as a valuable comparator, illustrating the complex evolutionary dynamics within *V. fischeri* clades.

## ANNOUNCEMENT

**Fig 1:**
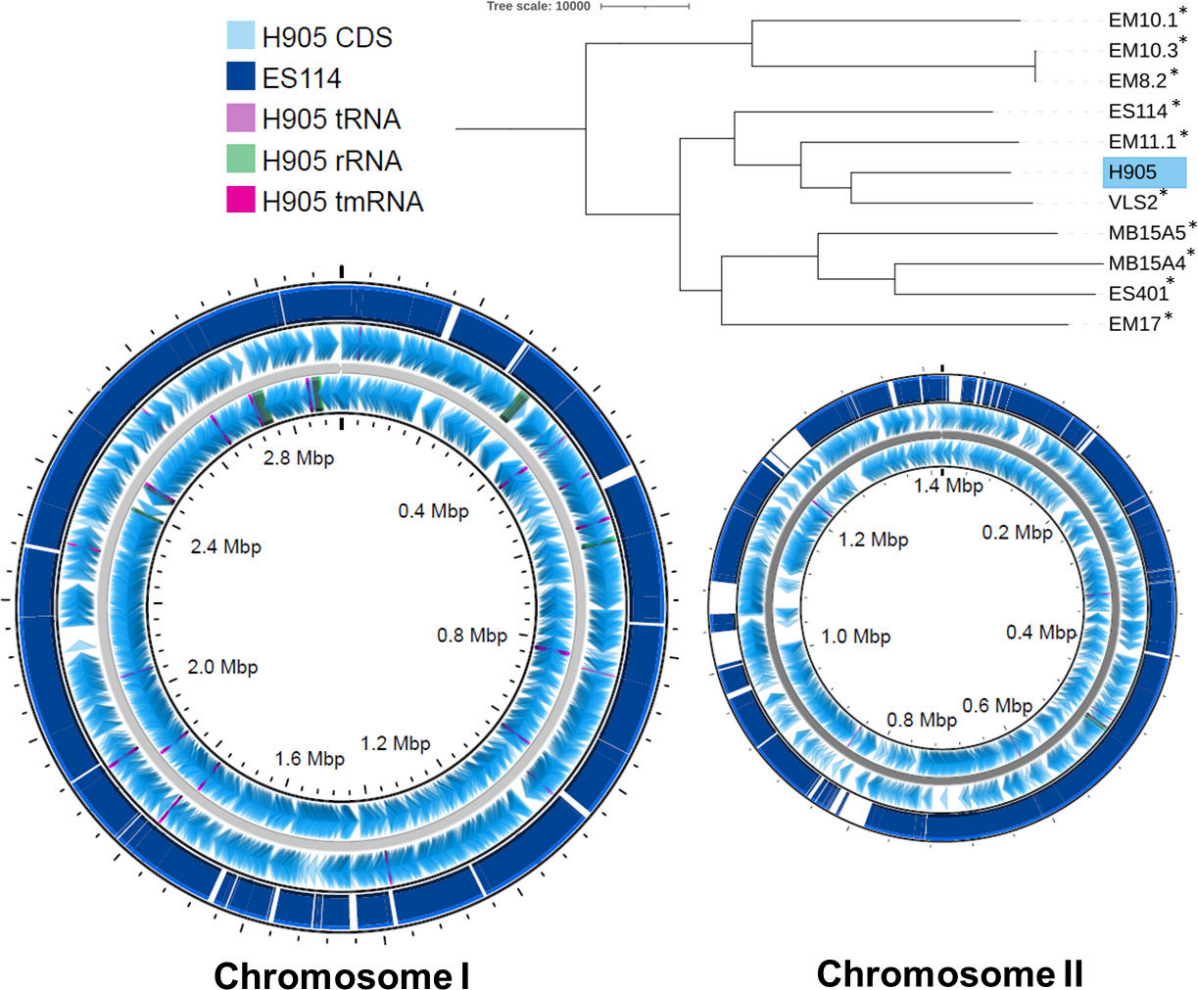
Strain H905 is closely related to and collinear with strain ES114. (A). H905 and ES114 form a clade along with light organ isolates VLS2 and EM11.1. Asterisks indicate light organ/symbiotic isolates from *E. scolopes*. The phylogenetic tree was inferred using a generalized time reversible (GTR) and gamma models in RAxML with 1,000 bootstraps (17). (B). H905 is collinear with ES114. Dark blue regions indicate shared genomic content. Sequence comparison performed with BLAST version 2.12.0 in Proksee (5).

*Vibrio* (*Aliivibrio*) *fischeri* (1) is a bioluminescent bacterium known for its mutualism with the Hawaiian bobtail squid, *Euprymna scolopes* (2). Symbiotic capacity does not correlate with phylogeny, making *V. fischeri* a model to study evolutionary dynamics underlying symbioses and sympatric evolution. Here we report the genome sequence of *V. fischeri* strain H905, a planktonic isolate from the *E. scolopes* habitat (3). H905 does not competitively colonize *E. scolopes* despite its cladal relationship with squid isolates, including strain ES114 (Fig. 1; 2, 4) . This genome will aid in identifying variation that could explain divergent features.

H905 was isolated from seawater at Kaneohe Bay, Oahu, Hawaii, in 1990, and its taxonomy was previously determined (3). DNA was extracted from Luria-Bertani + salt (LBS) broth cultures (6) inoculated with a colony isolated on LBS agar from a cryopreserved stock maintained at the University of New Hampshire using the Promega Wizard Genomic DNA Purification Kit (Madison, WI, USA) for Illumina sequencing and using an alkaline lysis/detergent protocol, followed by organic extraction to produce high-molecular-weight DNA (7) for Pacific Biosciences sequencing. Library preparation and sequencing using the Pacific Biosciences platform were per manufacturer’s instructions and reagents (Pacific Biosciences, Menlo Park, CA, USA) unless noted using the P5-C3 chemistry at the Icahn School of Medicine as previously detailed (8). DNA was sheared to ~20,000 bp by 2 × 60 s, 4,500 rpm spins in a G-tube column (Covaris, Woburn, MA, USA), purified twice with 0.45× AMPure XP (Beckman Coulter, Indianapolis, IN, USA), and repaired in DNA damage repair solution. SMRTbell adapters were end-ligated at 25°C overnight, and un-ligated fragments were enzymatically removed. Following size selection to 7,000–50,000 bp (0.75%; Sage Science Blue Pippin, Beverly, MA, USA), the primer was annealed at 80°C for 90 s, and temperature decreased by 0.1°/s to 25°C. The polymerase-template complex was bound for 4 hours at 30°C before loading on magnetic beads at 4°C for 60 minutes and placing onto the RSII machine at ~150 pM, configured for 180-minute continuous sequencing. Libraries for Illumina Hi-Seq 2500 (150 PE) sequencing were prepared with a modified Nextera protocol (9) and sequenced at the University of New Hampshire Hubbard Genome Center.

The H905 genome was assembled *de novo*, corrected, and trimmed from a total of 28,151 long reads (average length 2,957 bp, N50 = 3704) using CANU version 1.6 (10). The assembly generated three contigs consisting of two chromosomes and one phage when the corMhapSensitivity setting was adjusted to high. Overhangs were identified and trimmed using Circlator version 1.5.5 (11). Chromosomes were oriented according to genome ES114 (accession numbers CP000020, CP000021, CP000022) (2), and the phage was oriented via Prodigal (12). Adapter trimmed (13) Illumina reads (11,991,486 paired reads, average read length 101 bp) were aligned to the Pacific Biosciences assembly using Burrows-Wheeler alignment tool (BWA) (14) and indexed using SAMtools (15), and errors were corrected using Pilon version 1.20 (16). The final genome coverage was 2,802×. The H905 genome is 4.4 Mbp with a GC content of 38.9% and 4,180 predicted genes and is collinear with ES114 (Fig. 1; 17). Default parameters were used for all software programs except where noted.

## Data Availability

Data are available at the National Center for Biotechnology Information and identified as *Aliivibrio fischeri* under the following accession numbers: SRR28533042 for Pacific Biosciences raw reads, SRR28602613 for Illumina raw reads, CP160629 for chromosome I and CP160630 for chromosome II for the hybrid assembly, and PP986400 for phage vB_Alfi_H905. This is version 1 of the complete genome assembly.
